# Gender and ethnic diversity in global ophthalmology and optometry association leadership: a time for change

**DOI:** 10.1111/opo.12793

**Published:** 2021-03-02

**Authors:** Aryati Yashadhana, Neriah A Clarke, Justine H Zhang, Jawad Ahmad, Shaffi Mdala, Priya Morjaria, Miho Yoshizaki, Fatima Kyari, Matthew J Burton, Jacqueline Ramke

**Affiliations:** 1 School of Public Health & Community Medicine, University of New South Wales, Sydney, Australia; 2 Centre for Health Equity Research Training & Evaluation (CHETRE), University of New South Wales, Sydney, Australia; 3 School of Social Sciences, University of New South Wales, Sydney, Australia; 4 University of Birmingham Medical School, Birmingham, UK; 5 International Centre for Eye Health, London School of Hygiene & Tropical Medicine, London, UK; 6 Manchester Royal Eye Hospital, Manchester, UK; 7 Ophthalmology Department, Queen Elizabeth Central Hospital, Blantyre, Malawi; 8 College of Health Sciences, University of Abuja, Abuja, Nigeria; 9 Moorfields Eye Hospital, London, UK; 10 School of Optometry and Vision Science, University of Auckland, Auckland, New Zealand

**Keywords:** optometry, ophthalmology, leadership, equity, gender, ethnic diversity, eye health

## Abstract

**Purpose:**

To assess the diversity of leadership bodies of member organisations of the International Council of Ophthalmology (ICO) and the World Council of Optometry (WCO) in terms of: (1) the proportion who are women in all world regions, and (2) the proportion who are ethnic minority women and men in Eurocentric high-income regions.

**Methods:**

We undertook a cross-sectional study of board members and chairs of ICO and WCO member organisations using a desk-based assessment of member organisation websites during February and March 2020. Gender and ethnicity of board members and chairs were collected using a combination of validated algorithmic software and manual assessment, based on names and photographs where available. Gender proportions were calculated across Global Burden of Disease super-regions, and gender and ethnicity proportions in the high-income regions of Australasia, North America and Western Europe.

**Results:**

Globally, approximately one in three board members were women for both ICO (34%) and WCO (35%) members, and one in three ICO (32%) and one in five WCO (22%) chairpersons were women. Women held at least 50% of posts in only three of the 26 (12%) leadership structures assessed; these were based in Latin America and the Caribbean (59% of WCO board positions held by women, and 56% of WCO chairs), and Southeast Asia, East Asia and Oceania (55% of ICO chairs). In the Eurocentric high-income regions, white men held more than half of all board (56%) and chair (58%) positions and white women held a further quarter of positions (26% of board and 27% of chair positions). Ethnic minority women held the fewest number of board (6%) and chair (7%) positions.

**Conclusions:**

Improvements in gender parity are needed in member organisations of the WCO and ICO across all world regions. In high-income regions, efforts to address inequity at the intersection of gender and ethnicity are also needed. Potential strategies to enable inclusive leadership must be centred on structurally enabled diversity and inclusion goals to support the professional progression of women, and people from ethnic minorities in global optometry and ophthalmology.

## Introduction

Equality for women and ethnic minorities in ophthalmic and optometric employment,^1,2^ clinical practice^3^ and academia^4^ is yet to be achieved. This is partly attributable to a lack of female representation in Science, Technology, Engineering, Maths and Medicine (STEMM) professions broadly.^5^ It is also underpinned by a pervasive construct of gender and race that socially categorises women and ethnic minorities in STEMM as less competent, allowing the reinforcement of stereotypes that reproduce workplace discrimination, unequal pay and a lack of parity among leadership positions.^6^

Diversity within organisational leadership has been shown to promote gender and ethnic-minority focused agendas and social responsiveness.^7^ Fostering equitable, diverse and inclusive representation among global eye health leaders is critical, in order to adequately respond to the complexities of global eye health inequity. Those in leadership roles have the power to change and influence structures and behaviour. For this reason, inclusion and diversity of leadership in Global Eye Health is a theme of the *Lancet Global Health* Commission on Global Eye Health.^8^ To inform the Commission, we have undertaken several studies to provide a baseline to track and propel change.^9^ In the study presented here, we aimed to document the inclusion of women and people from ethnic minority backgrounds in global ophthalmology and optometry leadership by assessing the governing bodies of member organisations of the International Council of Ophthalmology (ICO) and the World Council of Optometry (WCO).

## Methods

This is a desk-based, cross-sectional study. The sample frame was all member organisations listed on the websites of ICO and WCO on 10 February 2020. These lists were downloaded and during February and March 2020, we attempted to locate the website of each organisation. We assigned each organisation to the relevant Global Burden of Disease (GBD) super-region: High-income, Latin America & Caribbean, Sub-Saharan Africa, North Africa & Middle-East, South Asia, Southeast Asia/East Asia & Oceania and Central Europe/Eastern Europe & Central Asia.

From each website we extracted all available names of the members of the organisation’s governing body (hereafter referred to as the board), including the chairperson. Where a gender pronoun, gendered name (e.g., John = male) or photo for the board member was available, we assessed and manually recorded their gender, otherwise we recorded it as ‘unknown’. For board members of organisations located in the GBD high-income regions with predominantly European populations (Australasia, North America and Western Europe) we also assessed ethnic minority status from available photos, or otherwise recorded it as ‘unknown’.

We also used validated software tools to assign gender (Gender-API, version 3.14; www.gender-api.com) and ethnicity (Onolytics, 2020 version; www.onolytics.com) to each person based on their name. Gender-API contained 3,216,769 validated names from 191 countries (December 2020); in 2018 it was assessed as the best-performing name-to gender inference software in terms of the lowest proportion of inaccuracies (7.9%) and non-classifications (3.0%), although through a small proportion of misclassification, it may underestimate the proportion female.^10^ Further, Onolytics has been validated and widely used for assigning ethnicity.^11–13^ We compared these results with our manual assessment. In the event of a discrepancy, the assessment of the photograph was used and when this was unavailable, the algorithm result was used. When the algorithm and our assessment both resulted in an ‘unknown’ status we attempted a Google search of the individual to find a photograph; if no photo was obtained these are reported as missing data.

We calculated the proportion of board members and chairpersons who were women across each GBD super-region. For organisations based in Australasia, North America and Western Europe, we calculated the proportion of position holders who were women and men from an ethnic minority.

## Results

We located and extracted data on governance structures from websites of 123/177 ICO and 46/53 WCO organisations (*Table**1*). We were unable to identify websites for the remaining organisations, or the information was unavailable on the website. The number of organisations within a GBD super-region ranged from zero WCO members in Central Europe, Eastern Europe and Central Asia, up to 60 ICO members in the High-income Country super-region (*Table* *1*).

**Table 1:** Proportion of boards and chairs of member organisations of the World Council of Optometry (WCO) and the International Council of Ophthalmology (ICO) who are women, 2020. GBD = Global Burden of Disease

GBD Super-regions	Organisations		Board Members		Chairperson	
	Data available*n*	No data available^a^*n*	Female*n* (%)	Total^b^*n*	Female*n* (%)	Total^c^*n*
WCO						
High-income^d^	16	4	46 (28.9)	159	3 (18.8)	16
Latin America & Caribbean	9	—	43 (58.9)	73	5 (55.6)	9
North Africa & Middle East	6	1	3 (11.5)	26	—	7
South Asia	3	—	8 (30.8)	26	—	2
South-East Asia, East Asia & Oceania	4	—	10 (34.5)	29	1 (25.0)	4
Sub-Saharan Africa	8	2	16 (32.7)	49	1 (12.5)	8
Total	46	7	126 (34.8)	362	10 (21.7)	46
ICO						
Central Europe, Eastern Europe and Central Asia	12	11	51 (38.1)	134	1 (9.1)	11
High-income^d^	60	10	160 (31.6)	507	20 (34.5)	58
Latin America & Caribbean	13	4	67 (43.2)	155	5 (35.7)	14
North Africa & Middle East	12	6	22 (21.6)	102	6 (37.5)	16
South Asia	6	—	9 (19.1)	47	—	6
South-East Asia, East Asia & Oceania	12	4	61 (38.4)	159	6 (54.5)	11
Sub-Saharan Africa	8	19	16 (37.2)	43	2 (25.0)	8
Total	123	54	386 (33.7)	1,147	40 (32.3)	124

^a^Websites did not exist, did not have information about leadership or were unable to be translated.

^b^Six board members of ICO organisations had an unknown gender and were excluded.

^c^Some organisations did not report a chair and some reported ≥ 2 co-chairs.

^d^Includes organisations from the regions: Asia Pacific high-income, Australasia, Southern Latin America, North America and Western Europe.

The gender algorithm was able to classify 96.6% of the 1,515 names in our database and the ethnicity algorithm classified 97.0% of the 568 names in the three regions assessed. Our manual assessment agreed with the software in 95% and 93% of cases for gender and ethnicity, respectively, and, using either method, only six people were unable to be assigned a gender and four people were not assigned ethnicity status.

Globally, women held approximately one-third of board member positions for ICO (33.7%) and WCO (34.8%) organisations. Women were also the chairperson of one-third of ICO organisations (32.3%) but only one-fifth of WCO organisations (21.7%), see *Table* *1*. Across regions there was large variation. Latin America & the Caribbean was the region in which women’s participation in boards was highest (43.2% for ICO members and 58.9% for WCO members) while North Africa & Middle East and South Asia were the regions with lowest female participation in boards and chairpersonships (Table *1*).

In the high-income regions of Australasia, North America and Western Europe, white/non-ethnic minority men held more than half of all board memberships (56.0%) and chair positions (58.3%) and white/non-ethnic minority women held a further quarter of positions (25.9% of board memberships and 26.7% of chairs) (*Table* *2*). Ethnic minority women were the group with the lowest board membership across all three regions and held 6.0% of board memberships overall; they also held the fewest chair positions overall (*n* = 4, 6.7%). Ethnic minority men fared better than ethnic minority women in all positions except for chair positions in North America (one man compared to two women were chairs) (*Table**2*).

**Table 2:** Board members and chairpersons of 60^a^ member organisations of the International Council of Ophthalmology (*n* = 48) or the World Council of Optometry (*n* = 12) in Australasia, North America and Western Europe disaggregated by gender and ethnic minority status, 2020

Region	Non-ethnic minority men		Ethnic minority men		Non-ethnic minority women		Ethnic minority women	
	*n*	row %	*n*	row %	*n*	row %	*n*	row %
Australasia								
Chair	1	33.3	1	33.3	1	33.3	—	—
Board members	20	52.6	8	21.1	7	18.4	3	7.9
North America								
Chair	13	52.0	1	4.0	9	36.0	2	8.0
Board members^b^	129	49.2	39	14.9	71	27.1	23	8.8
Western Europe								
Chair	21	65.6	3	9.4	6	18.8	2	6.3
Board members^c^	166	63.1	21	8.0	68	25.9	8	3.0
All three regions								
Chair	35	58.3	5	8.3	16	26.7	4	6.7
Board members	315	56.0	68	12.1	146	25.9	34	6.0

^a^Organisations in high-income regions of Southern Latin America and Asia Pacific high income not included (*n* = 14); not all organisations had chairperson information available.

^b^Excludes one person with unknown ethnicity.

^c^Excludes two persons with unknown ethnicity; one person with unknown gender and one person with ethnicity and gender both unknown.

## Discussion

These results highlight the issue of gender equity and ethnic diversity within global ophthalmology and optometry leadership, with one in three board members being women for both ICO and WCO members, and one in three (ICO) or one in five (WCO) chair positions being held by women. Our analysis in Australasia, North America and Western Europe also points to the importance of considering inequity at the intersection of gender and ethnicity, with white men almost universally holding a greater proportion of leadership positions than the three other groups combined (*Table* *2**)*. The particularly sparse representation of ethnic minority women at the ICO and WCO board and chair level echoes the similar results our group recently reported on member organisations of the International Agency for the Prevention of Blindness.^9^

The lack of diversity in STEMM globally is often ascribed to a pipeline problem, which may contribute to some of our findings. For example, despite one-third of the American population identifying as an ethnic minority, only 6% of practicing ophthalmologists and ophthalmology faculty are from an ethnic minority background.^1^ Similarly, in Aotearoa, New Zealand, 5% of ophthalmologists identified as Māori or Pasifika in 2017, despite these groups comprising almost one-quarter of the population.^14^ Women are also underrepresented in ophthalmology, with recent reports from the USA and Australia showing women were 23% and 21% of practicing ophthalmologists, respectively.^1,15^ However, efforts to address this imbalance are evident with women being 45% and 48% of trainees in these two countries.^1,16^ Unfortunately, gender-disaggregated data on practicing or training clinicians are unavailable from many countries, so the extent to which gender-equity is improving in regions such as Sub-Saharan Africa is unknown.

In contrast, the pipeline cannot fully explain the results for optometry, particularly in many high-income countries where it is a predominantly female profession.^17,18^ Instead, the underrepresentation of women in optometry leadership may reflect gendered pressures such as the greater domestic responsibilities women provide,^19^ leading to less time available to take on additional professional responsibilities. Fewer women in leadership roles may also be due to differential attainment and career progression, including male employees being rewarded and promoted over equally qualified females.^6,20,21^ This was highlighted in a recent study investigating gender parity within the editorial boards of high impact ophthalmic journals in 2019, which reported poor representation of women, particularly the absence of female Editors-in-Chief.^20,22^ The lower number of female ophthalmologists partly explains this (a pipeline problem), but evidence suggests that significantly higher levels of productivity by women may be necessary to achieve parity in ophthalmic leadership positions.^20^

The benefits of a more diverse eye care workforce are many. Clinicians from ethnically diverse backgrounds are well equipped to provide culturally safe care, as racial-ethnic and language concordance with their patients contributes to increased patient trust and satisfaction, and better continuity of care. Indeed, in Australia, the training and employment of an Indigenous eye care workforce has been identified as key to reducing the pervasive inequity in eye health outcomes between Indigenous and non-Indigenous Australians.^23^ Additionally, ethnically diverse practitioners are more likely to work in underserved communities, and conduct research to address inequity.^1,24^ As stated above, leadership entities must also increase diversity to ensure that more socially responsive agendas are set. A further benefit of more women and ethnic minority people in leadership positions is the potential to increase interest in entering the profession, as these leaders can be role models for younger clinicians, and also tend to take on mentoring roles to support the progression of others.^24^

There is increasing recognition of the need to attract and support women and people from ethnic minorities into ophthalmology to better reflect the population,^25^ including global initiatives such as Women in Ophthalmology^26^ that aims to improve the professional environment for women, and national programmes such as the Minority Ophthalmology Mentoring Program and the RANZCO Māori Action Plan.^16,24^ Further, ICO’s World Ophthalmology Leaders Program^27^ recognises the need to encourage and facilitate women and ethnic minorities to enter leadership roles. Similar examples were difficult to identify for optometry, and there is a lack of data from low- and middle-income countries. In many high-income countries, the profession is becoming more female, with women being the majority of optometry students graduating each year. This shift creates more women available to take on leadership roles in the years ahead, but in isolation may be insufficient to achieve gender parity.

To support the sustained progression of women in optometry and ophthalmology, we believe strategies should be centred on structurally enabled diversity and inclusion goals, with a focus on women with intersectional identities—such as ethnicity, class or sexuality—that are often devalued by the dominant culture.^28,29^ One such strategy is for ICO and WCO to develop and promote guidelines for their member organisations that mandate equity, diversity and inclusion targets. They could then undertake annual benchmarking similar to the Global Health 5050 consortium which demonstrated rapid improvement is possible—one year after their baseline assessment, the proportion of board chairs of the consortium’s members increased from 25% to 35%.^28^ Another strategy would be to sufficiently resource exercises that monitor the profession nationally and globally^30^ to ensure data are disaggregated by gender and ethnic minority status (where relevant).

Creating a global eye health environment where women, people from ethnic minorities and low- and middle-income countries feel valued is essential to advance an equitable global eye health agenda.^9^ A strategy to enable this could involve a requirement for potential leaders to undertake training on unconscious bias and cultural safety prior to taking up their post and demonstrate a plan to address their own prejudices and biases. A recent example from Australasia demonstrates that structural change is possible.^31^ Because men—particularly white men in high-income countries—are overrepresented in optometric and ophthalmic leadership, we believe they must take a lead role in advocating for change, in order for gender and racial/ethnic equity to be accepted and realised in the global eye health sector.^32^

We believe this is the first attempt to assess the diversity of leadership of all ICO and WCO organisations. Our results must be interpreted in the context of several limitations. First, one in three ICO and one in six WCO member organisations did not have accessible information on their leadership structure. The organisations we could not access were disproportionately in Sub-Saharan Africa and Central Europe, Eastern Europe and Central Asia and it is unclear whether the leadership diversity of these organisations would differ substantially from included organisations. Second, we were unable to assess whether the information available on websites was up-to-date. Third, both the software and our assessment of photographs may not have accurately determined how a person identifies in terms of gender and ethnicity status. Gender-API may slightly underestimate the amount of females,^10^ but not to an extent that would diminish the gender-gap observed. We also acknowledge that this gender-binary approach fails to reflect individuals who do not identify as women or men. Fourth, while we have assessed diversity, we were unable to assess the extent to which women and people from ethnic minorities feel included and empowered in the leadership structures in which they are members. Finally, we recognise this global analysis prohibits a nuanced understanding of the situation within each member organisation, and initiatives may be underway. Regardless, as seen in the Global Health 5050 example outlined above, we believe a baseline assessment of this nature can provide a powerful catalyst for action for organisations not yet addressing equity, diversity and inclusion in their leadership structures, as well as reinforcement for those organisations attempting to create this positive change.

In the World Report on Vision, WHO highlighted the need for inclusive and participatory leadership to deliver Universal Health Coverage for eye health.^33^ Our results reinforce that power structures within regional and national organisations must be addressed to ensure a broader range of views are represented within leadership groups and a more responsive agenda can be set.^29^ We must continue to increase numbers of women and people from ethnic minority backgrounds in optometry and ophthalmology so that the profession better reflects the population they serve.^25,34^ In order to realise equity in eye health, we must also challenge and change the normative constructs of power, gender and ethnicity that continue to prevent the progression of women and ethnic minorities to attain positions of leadership in global eye health.

We hope that the World Report on Vision and the *Lancet Global Health* Commission on Global Eye Health can provide the impetus to create and sustain meaningful change globally and nationally, including for equity in eye health. We believe equity and inclusion in all eye health leadership bodies is critical for equity in eye health to be realised, and commend all organisations attempting to enable these changes. We challenge ICO, WCO and its member organisations to ensure that a repeat of this exercise in the coming years will find leadership structures that are more equitable, diverse and inclusive.
